# Weight Regain after Bariatric Surgery

**DOI:** 10.3390/jcm12093265

**Published:** 2023-05-04

**Authors:** Lara Ribeiro-Parenti, Clement Baratte, Tigran Poghosyan

**Affiliations:** 1 Service de Chirurgie Digestive, Œsogastrique et Bariatrique, Hôpital Bichat—Claude-Bernard, Assistance Publique-Hôpitaux de Paris, 75018 Paris, France; 2 UMR-S 1149 Centre de Recherche sur l’Inflammation, INSERM, 75018 Paris, France; 3 Université Paris Cité, 75006 Paris, France

Bariatric surgery (BS) is currently the most effective treatment for obesity, with long-lasting weight loss and improvement of related co-morbidities. BS is considered successful mainly based on resolution of co-morbidities and improvement of quality of life.

Unlike weight loss outcomes in BS (mean BMI of the cohort, change in BMI, percent of total weight loss (%TWL), percent excess BMI loss (%EBMIL) and/or percent excess weight loss (%EWL) in which ideal weight is defined by the weight corresponding to a BMI of 25 kg/m^2^) [1], weight regain (WR) is not well defined. The weight nadir is mostly reached within 12 to 18 months after surgery, followed by weight stabilization [2]. Weight regain, which is different from insufficient weight loss, can be observed after any type of bariatric surgery [3]; however, WR rate may be lower after gastric bypass than after sleeve gastrectomy [4]. 

WR after bariatric surgery is a serious issue, since it is often associated with comorbidity recurrence, and deterioration of both quality of life and self-esteem. WR is often poorly reported due to a lack of very long-term follow-up sessions but also for lack of standardized definitions. Lauti et al. [5] reported rates ranging from 9 to 91% just by using different WR definitions on the same cohort.

Exploration of WR cases is mandatory. Surgical factors are often debated: a small gastric volume appears to be a key factor to reach and maintain long-term weight loss [6]. Therefore, in the case of sleeve gastrectomy, bougie size should range from 32 to 36 F [7]. The distance between the pylorus and the first staple line should be as short as possible to have effective gastric emptying and a minimal reservoir. Secondary gastric dilatation after bariatric surgery seems to influence WR, especially after gastric bypass but also after sleeve gastrectomy [8,9]. Except in case of gastrojejunal anastomotic stricture or gastric stenosis, we question whether dilatation is a consequence or cause of eating disorders.

Several eating disorders, such as grazing [10], binge eating, loss of control, and night eating syndrome, have been reported after different bariatric procedures [11] and can lead to WR. Changes in eating behaviors must be accompanied with regular physical activity for long-lasting weight loss.

Patient’s compliance to care is also a corner stone of weight maintenance. Unfortunately, only a few patients follow the dietary and physical activity recommendations [12], even in the short term, or go to post-operative visits. The lack of follow-up session may also be associated with WR [13].

Intestinal adaptation after BS can also lead to WR. It is known that the release and response to gastro-intestinal or adipogenic hormones, such as ghrelin, glucagon-like peptide 1, peptide YY, and leptin, change after bariatric surgery which could participate in WR [14].

Currently, 10 to 20% of patients undergo revisional surgery for WR [15].

The results of revisional surgery, such as gastric pouch resizing or changes to bile or alimentary loop lengths, are relatively disappointing with variable weight loss results. The promising role of GLP-1 agonists in the management of WR after bariatric surgery has to be assessed on a wider scale [16,17].

As WR is multifactorial, everyone has a key role: surgeons must perform a perfect procedure that best suits the patient. Patients must comply with post-operative recommendations and attend regular and long-term follow-up sessions, as prevention is better than curative. The place of drug therapy must be specified. Revisional surgery should be at last resort. Furthermore, a universal definition of WR is needed in order to facilitate comparisons across studies and different surgical procedures.
